# Bioelectrical phase angle and psoriasis: a novel association with psoriasis severity, quality of life and metabolic syndrome

**DOI:** 10.1186/s12967-016-0889-6

**Published:** 2016-05-10

**Authors:** Luigi Barrea, Paolo Emidio Macchia, Carolina Di Somma, Maddalena Napolitano, Anna Balato, Andrea Falco, Maria Cristina Savanelli, Nicola Balato, Annamaria Colao, Silvia Savastano

**Affiliations:** I.O.S. & COLEMAN Srl, Naples, Italy; Dipartimento di Medicina Clinica e Chirurgia, Unit of Endocrinology, Federico II University Medical School of Naples, Via Sergio Pansini 5, 80131 Naples, Italy; IRCCS SDN, Napoli Via Gianturco 113, 80143 Naples, Italy; Dipartimento di Medicina Clinica e Chirurgia, Unit of Dermatology, Federico II University Medical School of Naples, Via Sergio Pansini 5, 80131 Naples, Italy

**Keywords:** Environmental factors, Phase angle (PhA), Psoriasis area and severity index (PASI) score, Dermatology life quality index (DLQI), Metabolic syndrome (MetS)

## Abstract

**Background:**

Obesity, metabolic syndrome (MetS), and psoriasis, largely driven by environmental factors, show multiple bidirectional associations, with important metabolic implications in psoriatic patients. Besides body mass index (BMI) as a measure of obesity, data on phase angle (PhA), a direct measure by bioelectrical impedance analysis (BIA), used as a marker of cellular health and a predictor of morbidity and mortality in various diseases, are still lacking in psoriasis. In this case–control, cross-sectional study, we investigated the PhA in 180 pairs of adult psoriatic patients and healthy controls, evaluating also the potential use of the PhA as marker of the clinical severity, the quality of life, and the presence of the MetS in psoriatic patients.

**Methods:**

Anthropometric measures, metabolic profile and bioelectrical variables were evaluated. The clinical severity was assessed by standardized psoriasis area and severity index (PASI) score and c-reactive protein (CRP) levels, and the quality of life was evaluated by dermatology life quality index (DLQI). MetS was diagnosed according to Adult Treatment Panel III.

**Results:**

Psoriatic patients presented smaller PhA (p < 0.001) and higher prevalence MetS compared with controls. The PhA was significantly associated with number of parameters of MetS in both groups (p < 0.001). After adjusting for BMI, this association remained significant in psoriatic patients only (p < 0.001). Among psoriatic patients, the PhA was the major index value for the diagnosis of MetS (OR 5.87, 95 % CI 5.07–6.79) and was inversely associated with both PASI score and DLQI, independently of BMI (p < 0.001). At multiple regression analysis, the PhA well predicted the PASI score and DLQI. Based on ROC curves, the most sensitive and specific cutoffs of PhA to predict the highest PASI score and the lowest DQLI were ≤4.8° and ≤4.9°, respectively.

**Conclusions:**

We reported that psoriatic patients presented small PhAs, with a novel association between PhA, clinical severity, quality of life in psoriatic patients, and MetS. Further studies are required to validate the PhA’s prognostic ability in assessing the clinical severity and MetS in psoriatic patients.

## Background

Among environmental factors, a growing body of evidence supports the presence of multiple bidirectional associations among obesity, metabolic syndrome (MetS), and psoriasis, a chronic, immune mediated inflammatory skin disease with a complex component background [1, 2]. The precise mechanisms linking obesity, MetS, and psoriasis remain to be defined, but inflammatory pathways mutual to both conditions are probably involved [3–6], with important metabolic implications in the prognosis of psoriatic patients [7]. In this context, early detection and treatment of the metabolic derangements associated to psoriasis might represent an important goal in the management of psoriatic patients [8]. In most of these studies, body mass index (BMI) has been employed as a measure of obesity. By contrast, only few studies have investigated the association between psoriasis and body composition [9]. In particular, relationships have been evidenced between body composition and the occurrence of psoriasis [10], the severity of the disease [11], or the response to treatment with anti-TNF-α agents [12]. Currently, measures of body composition are evaluated by bioelectrical impedance analysis (BIA). BIA is not however a direct method for assessment of body composition and its accuracy as an indicator of body composition could be hampered by an altered distribution of extra- and intra-cellular water (ECW and ICW) [13].

Differently from the other parameters obtained by BIA, the phase angle (PhA), a direct measure by BIA, is a rapid, easy and bloodless tool in clinical setting. The PhA represents either the reactance of tissues (Xc) associated with cellularity, cell size and integrity of the cell membrane, and the resistance (R) of tissues, which is dependent on lean tissue mass and tissue hydration [13–15]. It is well established that a decrease in PhA is consistent with cell death and reflects a breakdown of cell membranes, an expansion of the interstitial fluid space (volume) with a concomitant decrease in ICW resulting in an increased spacing among affected cells. On the other side, larger PhAs reflect higher quantities of intact cell membranes and lean body mass [16]. In healthy population, PhA is affected by a number of different factors, including age, sex, and BMI [17, 18]. In disease conditions, including MetS, PhA values are frequently lower than normal since either disease-specific parameters and disease-related inflammatory status may impair PhA [19]. In this regards, the use of the PhA has been recommended as a prognostic marker of mortality in various chronic diseases [20], including cancer, and is associated with risk of morbidity in diabetes [21] and obesity [22]. As long as we know, data on the risk of morbidity in psoriatic patients and the association between PhA and MetS the clinical setting of the chronic, systemic inflammatory status mutual to both conditions are still lacking.

Aim of this case–control, cross-sectional study was: (i) to investigate the differences in the PhA between patients affected with psoriasis and healthy controls; (ii) to evaluate the association of the PhA with the clinical severity of the disease, assessed by standardized psoriasis area and severity index (PASI) score and c-reactive protein (CRP) levels, and the quality of life, evaluated by dermatology life quality index (DLQI); (iii) to verify the potential usefulness of PhA as an early marker of the MetS associated to psoriasis.

## Methods

### Design and setting

This is a case–control, cross-sectional observational study carried out at the Department of Clinical Medicine and Surgery, *Unit of Endocrinology*, University Federico II, Naples (Italy). The work has been carried out in accordance with the Code of Ethics of the World Medical Association (Declaration of Helsinki) for experiments involving humans, and it has been approved by the Ethical Committee of the University of Naples “Federico II” Medical School. The purpose of the protocol was explained to both the patients and the healthy controls, and written informed consent was obtained.

### Population study

**Fig. 1:**
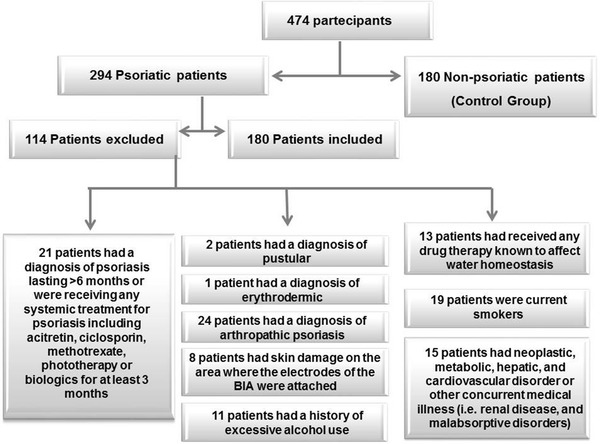
Flow chart of study design

The study has been conducted on 180 adult patients out of 294 Caucasian subjects of both gender affected by psoriasis attending the Psoriasis Care Center of the Outpatient Clinic of the *Section of Dermatology*, University of Naples Federico II from January 2014 to June 2015. In order to improve the power of the study, we increased the homogeneity of the patient sample by including adult treatment-naïve patients only, aged 18–65 years. The flow of study subjects is shown in Fig. 1. Patients were excluded from the study if they (1) had a diagnosis of psoriasis lasting >6 months or were receiving any systemic treatment for psoriasis including acitretin, ciclosporin, methotrexate, phototherapy or biologics for at least 3 months (21 patients); (2) had a diagnosis of pustular (2 patients), erythrodermic (1 patient) or arthropathic psoriasis (24 patients); (3) had skin damage on the area where the electrodes of the BIA were attached (8 patients); (4) had received any drug therapy known to affect water homeostasis (13 patients); (5) had a history of excessive alcohol use (11 patients); (6) were current smokers (19 patients); (7) had neoplastic, metabolic, hepatic, and cardiovascular disorder or other concurrent medical illness (i.e., renal disease, and malabsorptive disorders) (15 patients).

One hundred eighty non-psoriatic subjects were chosen as healthy controls among hospital volunteers and employees from the same geographical area. Controls were matched on the basis of age, sex and BMI. The exclusion criteria for controls were the same as the patients, with the additional criterion that controls had no previous diagnosis of psoriasis.

The PASI score is the gold standard for the severity assessment and widely used tool for measuring psoriasis severity [23]. The scale evaluates four areas of the body (head/neck, upper limbs, trunk, and lower limbs) for erythema, scaliness and thickness of psoriatic plaques. The PASI score can range from 0 to 72, with higher scores indicating greater severity. PASI score was used to be recorded by measuring four body surface areas (head, chest, upper and lower limbs) with patient in a standing position according to the method described by Harari M et al. 2000 [24] and the age at onset of it (early psoriasis: age <39 years and late psoriasis aged >40 years) were also noted. To prevent rate biases, the dermatologists who evaluated the PASI score were blinded to the design of the study. To assess the quality of life in our patients, we employed the dermatology life quality index (DLQI) [25]. The DLQI consists of ten questions organized in six sections. Those sections include symptoms and feelings (max. six points), daily activities (max. six points), leisure (max. six points), work and school (max. three points), personal relationships (max. six points) and treatment (max. three points). The achievable score ranges between 0 and 30 points with higher scores indicating a greater impact on quality of life.

### Anthropometric measurements

The measurements were made in a standard way by one operator (a nutritionist experienced in providing nutritional assessment and body composition).

All anthropometric measurements were taken with subjects wearing only light clothes and without shoes. In each subject, weight and height were measured to calculate the BMI [weight (kg) divided by height squared (m^2^), kg/m^2^]. Height was measured to the nearest 1 cm using a wall-mounted stadiometer. Body weight was determined to the nearest 50 g using a calibrated balance beam scale. Waist Circumference (WC) was measured to the closest 0.1 cm with a non-extensible tape at the natural indentation or at a midway level between the iliac crest and the lower edge of the rib cage if no natural indentation was visible. The measurement was made with the subject standing upright, feet together and arms hanging freely at the sides, with the subjects standing and breathing normally. The degree of obesity was established according to a scale based on BMI cut-off points: 35.0–39.9 (grade II obesity) and ≥40.0 kg/m^2^ (grade III obesity or severe obesity), respectively. Abdominal obesity was defined as: WC ≥102 cm in men and ≥88 cm in women. In all individuals were measured Systolic (SBP) and diastolic (DBP) blood pressure in three times, 2 min apart, with a random zero sphygmomanometer (Gelman Hawksley Ltd., Sussex, UK) after the subject had been sitting for at least 10 min. The average of the second and third reading was recorded.

Information on smoking habit, alcohol consumption, and physical activity was obtained by a standard questionnaire. Current smokers were defined as those who smoked at least one cigarette per day and former smokers as those who had stopped smoking more than 1 year before the interview; the rest of the participants were defined as noncurrent smokers. Participants were also classified according to their alcohol intake into two groups: at least one glass of wine (or an equivalent amount of other alcoholic beverages per day) (YES) or no alcohol consumption (NO). Physical activity level was expressed according to whether the participant habitually engaged at least 30 min/day of aerobic exercise (Yes/No).

### Metabolic syndrome and type 2 diabetes definitions

According to the NCEP ATP III definition, MetS is present if three or more of the following five criteria are met: WC ≥102 cm (men) or 88 cm (women), blood pressure ≥130/85 mmHg, fasting triglyceride level ≥150 mg/dl, fasting high-density lipoprotein (HDL) cholesterol level ≤40 mg/dl (men) or ≤50 mg/dl (women), and fasting glucose ≥100 mg/dl [26]. Type 2 diabetes (T2DM) was diagnosed according to ADA criteria [27].

### Bioelectrical impedance analysis

Bioelectrical impedance analysis was performed using a BIA phase-sensitive system by experienced observers (single-frequency 50 kHz BIA 101 RJL, Akern Bioresearch, Firenze) [28]. Based on the European Society of Parenteral and Enteral Nutrition (ESPEN) guidelines [29], all participants were supine with limbs slightly spread apart from the body, refrained from eating, drinking, and exercising for 6 h and no alcohol within 24 h before testing. Shoes and socks were removed and contact areas were scrubbed with alcohol immediately before electrode placement. Electrodes (BIATRODES Akern Srl; Florence, Italy) were placed proximal to the phalangeal–metacarpal joint on the dorsal surface of the right hand and distal to the transverse arch on the superior surface of the right foot. Sensor electrodes were placed at the midpoint between the distal prominence of the radius and ulna of the right wrist, and between the medial and lateral malleoli of the right ankle.

R (Ω, Ohm) and Xc (Ω, Ohm) were measured. The PhA was derived from conditions under 50 kHz according to the following formula: ${\text{PhA }}(^\circ ,{\text{ degrees}}) = {\text{arctangent Xc}}/{\text{R }}\left( {\left( {{{\text{Xc}} \mathord{\left/ {\vphantom {{\text{Xc}} {\text{R}}}} \right. \kern-0pt} {\text{R}}}} \right) \times \left( {{{180} \mathord{\left/ {\vphantom {{180} \pi }} \right. \kern-0pt} \pi }} \right)} \right)$.

### Biochemical measurements

Samples were collected in study population between 8 and 10 a.m. after an overnight fast of at least 8 h and stored at −80 °C until processed. All biochemical analyses including glucose, total cholesterol, triglycerides, aspartate aminotransferase (AST), and alanine aminotransferase (ALT) were performed with a Roche Modular Analytics System in the Central Biochemistry Laboratory of our Institution. Low-density lipoprotein (LDL) and HDL cholesterol were determined by direct method (homogeneous enzymatic assay for the direct quantitative determination of LDL and HDL cholesterol). CRP levels were determined with a nephelometric assay with CardioPhase high-sensitive from Siemens Healthcare Diagnostics (Marburg, Germany). The intra-assay coefficients of variations (CV) for CRP was <4 %; low detection limit was >0.1 mg/l.

### Statistical analysis

Results are expressed as mean ± SD or as median plus range according to variable distributions evaluated by Kolmogorov–Smirnov test (p < 0.01). To correct for skewed distributions, PASI score and DLQI were logarithmically transformed and back-transformed for presentation in text, tables and figures. Differences between psoriatic and healthy controls were analyzed by unpaired *t* test or Mann–Whitney *U*-test, as appropriate. The Chi square (χ^2^) test was used to determine the significance of differences in frequency distributions. The correlations between study variables were performed using Pearson *r* or Spearman’s *rho* correlation coefficients. Bivariate proportional odds ratio (OR) models were performed to assess the association among quantitative variables and qualitative variables (sex and presence/absence of MetS). Receiver operator characteristic (ROC) curve analysis was performed to determine sensitivity and specificity, area under the curve (AUC), and confidence intervals (CI), as well as cutoff values for PhA values in detecting the clinical severity and the quality of life of psoriatic patients. Test AUC for ROC analysis was performed. We want show that AUC resulted 0.957 for a particular test is significant from the null hypothesis value 0.5 (meaning no discriminating power), than we enter 0.957 for AUC ROC and 0.5 for null hypothesis values. For *α* level we selected 0.05 type I error and for *β* level we selected 0.20 type II error. Two multiple regression analysis models (stepwise method), expressed as *r*^2^, Beta (*β*) and *t*, with PhA as dependent variables were used to estimate the predictive value of: (i) BMI, MetS parameters, and PASI score; (ii) BMI, MetS parameters, and DLQI. In these analyses, we entered only those variables that had a *p* value <0.05 in the univariate analysis (partial correlation). To avoid multicollinearity, variables with a variance inflation factor (VIP) >10 were excluded. Values ≤5 % were considered statistically significant. Data were stored and analyzed using the MedCalc^®^ package (Version 12.3.0 1993- 2012 MedCalc Software bvba—MedCalc Software, Mariakerke, Belgium). Proportional odds model was carried out using the R Project for Statistical Computing 2014 (http://www.R-project.org).

## Results

**Fig. 2:**
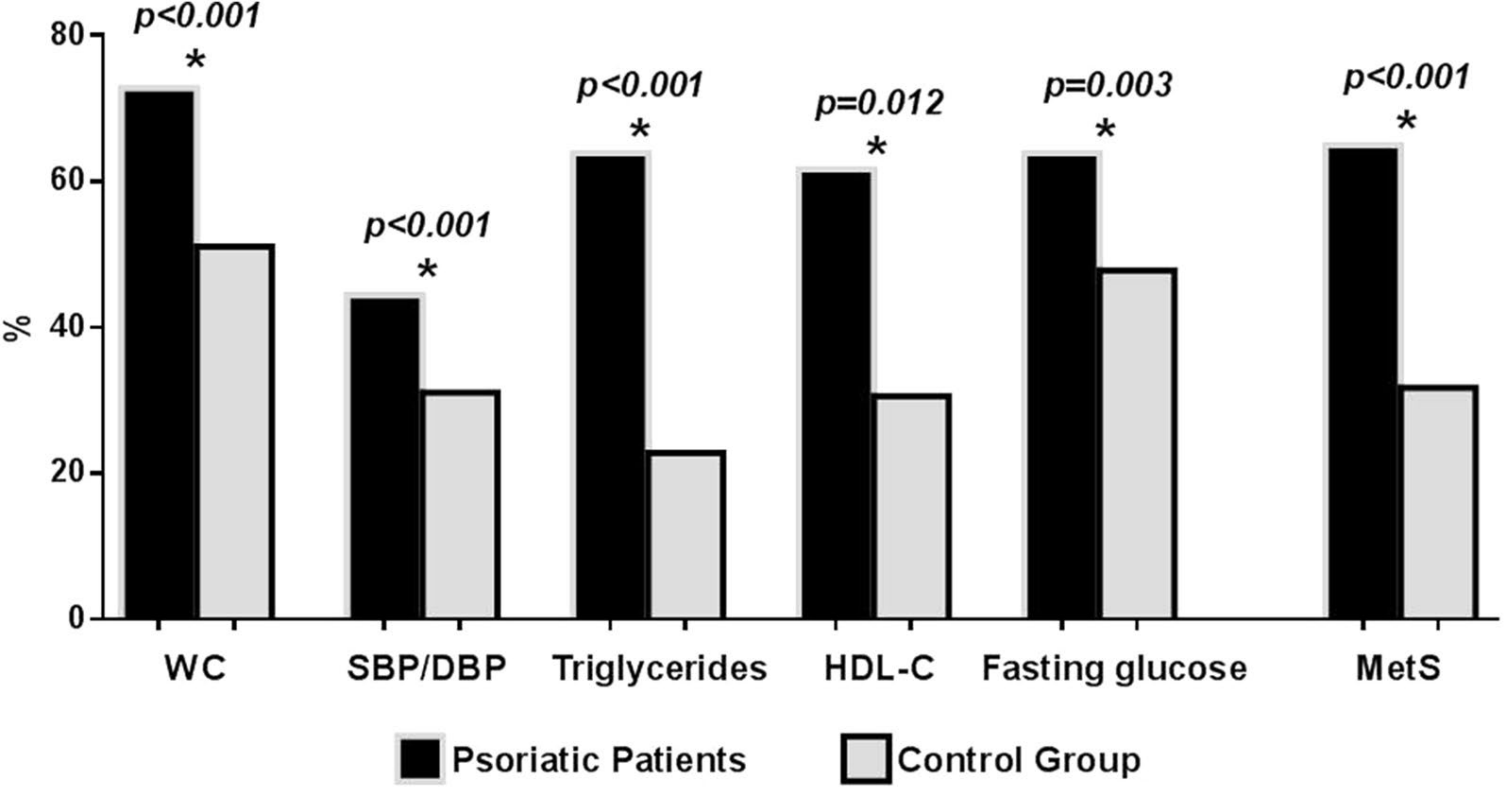
Frequency of metabolic risk factors and MetS in psoriatic patients and control group. The psoriatic patients exhibited statistically significant differences compared with controls for all the parameters of the MetS. In particular: WC (72.8 vs 51.1 %; χ^2^ = 17.0, p < 0.001), SBP/DBP (44.4 vs 31.1 %; χ^2^ = 6.3, p < 0.001), triglycerides (63.9 vs 22.8 %; χ^2^ = 60.3, p < 0.001), HDL-C (61.7 vs 30.6 %; χ^2^ = 33.8, p = 0.012); glucose (63.9 vs 47.8 %; χ^2^ = 8.8, p = 0.003) and MetS presence/absence (65.0 vs 31.7 %; χ^2^ = 38.7, p < 0.001); in psoriatic patients and control group, respectively. According to the NCEP ATP III definition, the MetS is defined as the coexistence of three or more of the following findings: (1) increased WC (≥102 cm for men and ≥88 cm for women), (2) hypertension (SBP/DBP ≥130/85 mmHg), (3) hypertriglyceridaemia (≥150 mg/dl), (4) low HDL cholesterol levels (≤40 mg/dl for men and ≤50 mg/dl for women) and (5) elevated fasting plasma glucose (≥100 mg/dl). Results are expressed as percentage. The Chi square (χ^2^) test was used to test the significance of differences between the two groups. A *p* value in bold type denotes a significant difference (p < 0.05). *WC* waist circumference, *SBP* systolic blood pressure, *DBP* diastolic blood pressure, *HDL*-*C* high-density lipoprotein cholesterol, *MetS* metabolic syndrome

**Fig. 3:**
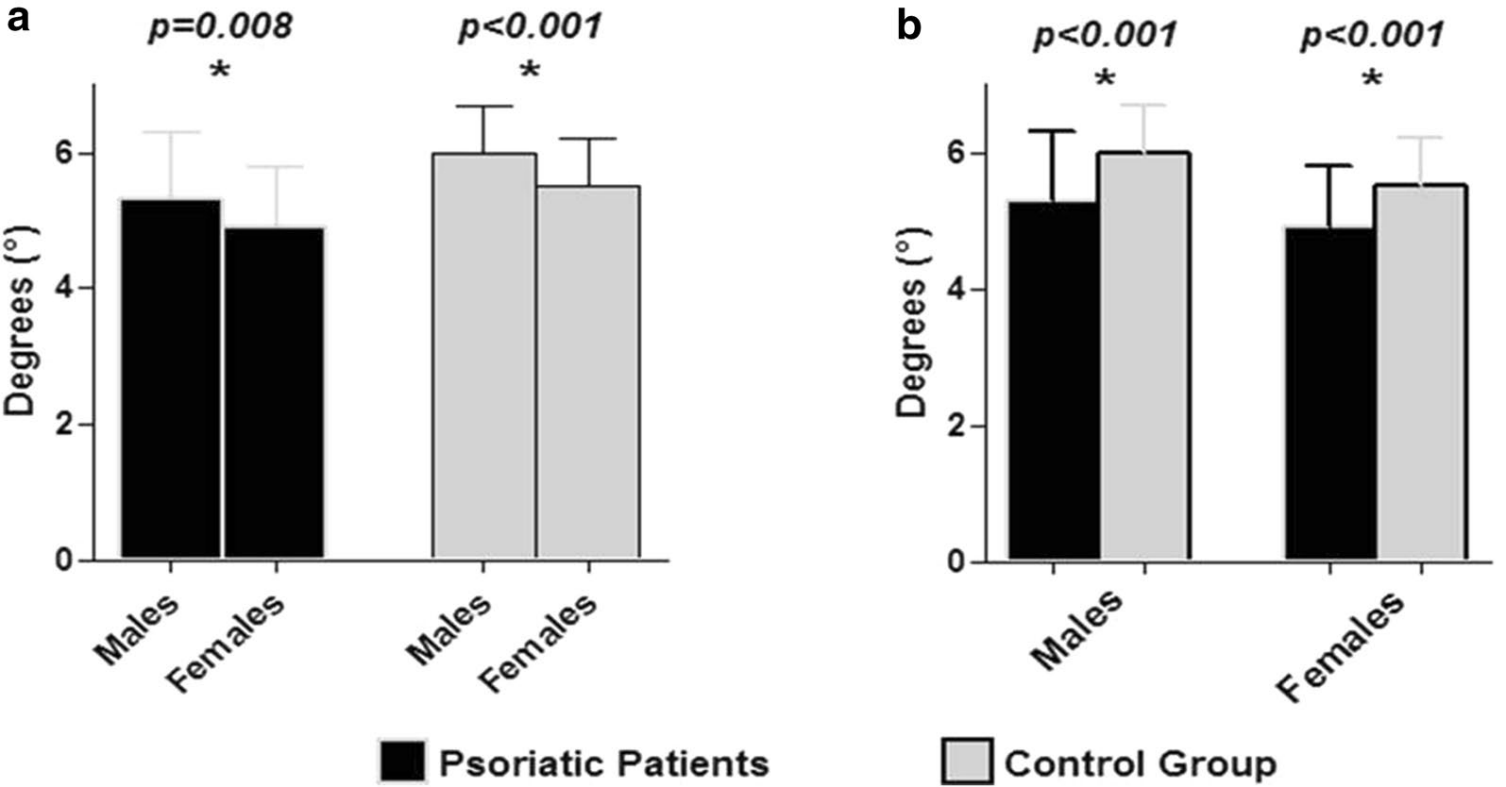
Difference in the PhA between psoriatic patients and control group according to gender. The gender-specific difference in the PhA between males and females was evidenced in psoriatic patients and control group (5.3 ± 1.0 vs 4.9 ± 0.9, p = 0.008 and 6.0 ± 0.7 vs 5.5 ± 0.7, p < 0.001; in males and females, respectively), (**a**) Although the PhA was smaller in males and females psoriatic patients than in controls, (5.3 ± 1.0 vs 6.0 ± 0.7, p < 0.001 and 4.9 ± 0.9 vs 5.5 ± 0.7, p < 0.001, respectively), (**b**). Results are expressed as mean ± SD according to variable distributions evaluated by Kolmogorov–Smirnov test. A *p* value in bold type denotes a significant difference (p < 0.05). Differences between groups were analyzed by paired *t* test

Study population consisted of 180 psoriatic patients, aged 21–65 years (71 % males), with BMI ranging 18.6–53.4 kg/m^2^. Control group consisted of 180 subjects, aged 29–65 years (71 % males), with BMI ranging 18.1–49.7 kg/m^2^. Among psoriatic patients, PASI score was 6.0 (0.20–28.80) and DLQI 1.3 (0.00–15.00). In psoriatic patients and healthy group the prevalence of current smoking was 36.7 vs 31.1 %, respectively (χ^2^ = 1.00; p = 0.316); the alcohol consumption was 28.9 vs 21.1 %, respectively (χ^2^ = 2.50; p = 0.114); while 33.3 vs 31.7 %, respectively (χ^2^ = 0.05; p = 0.822) reported having practiced physical activity. Anthropometry, metabolic profile and bioelectrical variables of both groups were summarized in Table 1. In both groups the majority of participants were overweight/obese (77.8 and 69.4 %, respectively; χ^2^ = 2.80; p = 0.094). Psoriatic patients presented a worse metabolic profile, a higher prevalence of T2DM (22.8 vs 9.4 %, respectively; χ^2^ = 10.87; p = 0.001), lower Xc, smaller PhA, and higher CRP levels (p < 0.001) compared with non-psoriatic counterparts. As reported in Fig. 2, MetS was more frequently diagnosed among psoriatic patients than in healthy subjects. The PhA was evaluated according gender in both psoriatic and healthy subjects. In particular, gender-specific differences in the PhA between males and females were evidenced in both in healthy subjects and psoriatic patients (p < 0.001 vs p = 0.008, respectively; Fig. 3a). However, compared with in healthy subjects, both males and females psoriatic patients had a smaller PhA (p < 0.001) (Fig. 3b).

**Table 1:** Descriptive and comparative statistics: psoriatic patient versus control group

Parameters	Psoriatic patients n = 180	Control group n = 180	*p* values
Age (years)	50 (21.0–65.0)	48.0 (29.0–65.0)	0.623
Anthropometric variables			
Weight (kg)	85.7 ± 20.1	84.1 ± 21.7	0.447
Height (m)	1.69 (1.49–1.92)	1.70 (1.50–1.85)	0.878
BMI (kg/m^2^)	30.2 ± 6.1	29.6 ± 7.3	0.422
Normal weight n (%)	40 (22.2 %)	55 (30.6 %)	0.094
Overweight n (%)	55 (30.6 %)	47 (26.1 %)	0.413
Obesity n (%)	85 (47.2 %)	78 (43.3 %)	0.525
WC (cm) males	108.8 ± 17.0	96.3 ± 21.5	*<0.001*
WC (cm) females	110.5 ± 26.3	95.2 ± 24.2	*0.002*
Metabolic profile			
SBP (mmHg)	130.0 (100.0–165.0)	125.0 (95.0–165.0)	*0.010*
DBP (mmHg)	80.0 (50.0–100.0)	75.0 (50.0–100.0)	*0.008*
Glucose (mg/dl)	105.0 (65.0–197.0)	96.0 (61.0–190.0)	*0.026*
Total cholesterol (mg/dl)	197.5 (97.0–315.0)	171.0 (102.0–332.0)	*0.025*
HDL cholesterol (mg/dl)	43.3 ± 11.0	45.7 ± 7.9	*0.023*
LDL cholesterol (mg/dl)	132.6 ± 44.7	121.7 ± 51.8	*0.049*
Triglycerides (mg/ml)	164.0 (36.0–402.0)	139.0 (66.0–273.0)	*0.009*
AST (U/l)	28.0 (10.0–94.0)	27.0 (5.0–65.0)	*0.013*
ALT (U/l)	29.0 (7.0–150.0)	25.0 (3.0–63.0)	*0.022*
CRP levels (ng/ml)	3.1 (0.1–16.2)	1.1 (0.0–3.9)	*<0.001*
Bioelectrical variables			
R (Ohm)	504.5 ± 85.8	492.9 ± 87.2	0.215
Xc (Ohm)	45.4 ± 9.2	50.1 ± 10.4	*<0.001*
PhA (°)	5.2 ± 1.0	5.8 ± 0.7	*<0.001*

Psoriatic patients exhibited statistically significant differences compared with controls for anthropometric measurements, metabolic profile and bioelectrical variables. Results are expressed as mean ± SD or as median plus range according to variable distributions evaluated by Kolmogorov–Smirnov test. A *p* value in italic denotes a significant difference (p < 0.05). Differences between groups were analyzed by paired *t* test or Wilcoxon signed-rank test, when appropriate

*BMI* body mass index, *WC* waist circumference, *SBP* systolic blood pressure, *DBP* diastolic blood pressure, *HDL* high-density lipoprotein, *LDL* low-density lipoprotein, *AST* aspartate aminotransferase, *ALT* alanine aminotransferase, *CRP* c-reactive protein, *R* resistance, *Xc* reactance, *PhA* phase angle

### Correlation studies

Correlations among PhA and study variables were analyzed in psoriatic patients and in healthy subjects separately, and reported in Table 2. The PhA showed significant negative associations with almost all the metabolic variables, although with some differences between the two groups. In particular, the PhA was significantly and negatively associated with age in healthy subjects only.

**Table 2:** Correlations among the PhA and study variables in psoriatic patient and control group

Parameters	Psoriatic patients				Control group			
	Simple correlation		Adjusted for BMI		Simple correlation		Adjusted for BMI	
	r	*p* value	r	*p* value	r	*p* value	r	*p* value
Age (years)	−0.098	0.191	−0.101	0.189	−0.790	*<0.001*	−0.709	*<0.001*
Anthropometric variables								
BMI (kg/m^2^)	−0.220	*0.003*	–	–	−0.551	*<0.001*	–	–
WC (cm)	−0.273	*<0.001*	−0.155	*0.045*	−0.537	*<0.001*	−0.112	0.156
Metabolic profile								
SBP (mmHg)	−0.119	0.112	−0.097	0.211	−0.259	*<0.001*	0.100	0.203
DBP (mmHg)	−0.125	0.094	−0.074	0.337	−0.311	*<0.001*	0.013	0.871
Glucose (mg/dl)	−0.027	0.721	0.001	0.990	−0.096	0.655	−0.049	0.539
Total cholesterol (mg/dl)	−0.261	*<0.001*	−0.331	*<0.001*	−0.332	*<0.001*	0.031	0.696
HDL cholesterol (mg/dl)	0.286	*<0.001*	0.247	*0.001*	0.269	*<0.001*	0.045	0.570
LDL cholesterol (mg/dl)	−0.331	*<0.001*	−0.303	*<0.001*	−0.315	*<0.001*	0.015	0.853
Triglycerides (mg/ml)	−0.368	*<0.001*	−0.343	*<0.001*	−0.148	*0.048*	0.060	0.447
AST (U/l)	−0.200	*0.007*	−0.183	*0.017*	−0.248	*0.041*	0.014	0.855
ALT (U/l)	−0.235	*0.001*	−0.127	0.099	−0.261	*<0.001*	0.162	*0.039*
MetS (n. parameters)	−0.380	*<0.001*	−0.334	*<0.001*	−0.425	*<0.001*	−0.020	0.805
CRP levels (ng/ml)	−0.320	*<0.001*	−0.283	*<0.001*	−0.522	*<0.001*	0.036	0.647
Bioelectrical variables								
R (Ohm)	−0.400	*<0.001*	−0.444	*<0.001*	−0.154	*0.039*	−0.069	0.385
Xc (Ohm)	0.625	*<0.001*	0.579	*<0.001*	0.516	*<0.001*	0.449	*<0.001*

Simple correlations and partial correlation adjusted for BMI among the PhA and anthropometric measurements, metabolic profile and bioelectrical variables. There was a significant negative association among the PhA and BMI, WC, lipid and hepatic profile, MetS and R and Xc; and significant positive association with HDL-cholesterol. In addition, after adjusting for BMI, the associations among the PhA, metabolic parameters and CRP levels remained still significant in psoriatic patients only

Correlations among variables were analyzed using Pearson *r* or Spearman’s *rho* correlation coefficients. A *p* value in italic denotes a significant difference (p < 0.05)

*PhA* phase angle, *BMI* body mass index, *WC* waist circumference, *SBP* systolic blood pressure, *DBP* diastolic blood pressure, *HDL* high-density lipoprotein, *LDL* low-density lipoprotein, *AST* aspartate aminotransferase, *ALT* alanine aminotransferase, *MetS* metabolic syndrome, *CRP* c-reactive protein, *R* resistance, *Xc* reactance

**Fig. 4:**
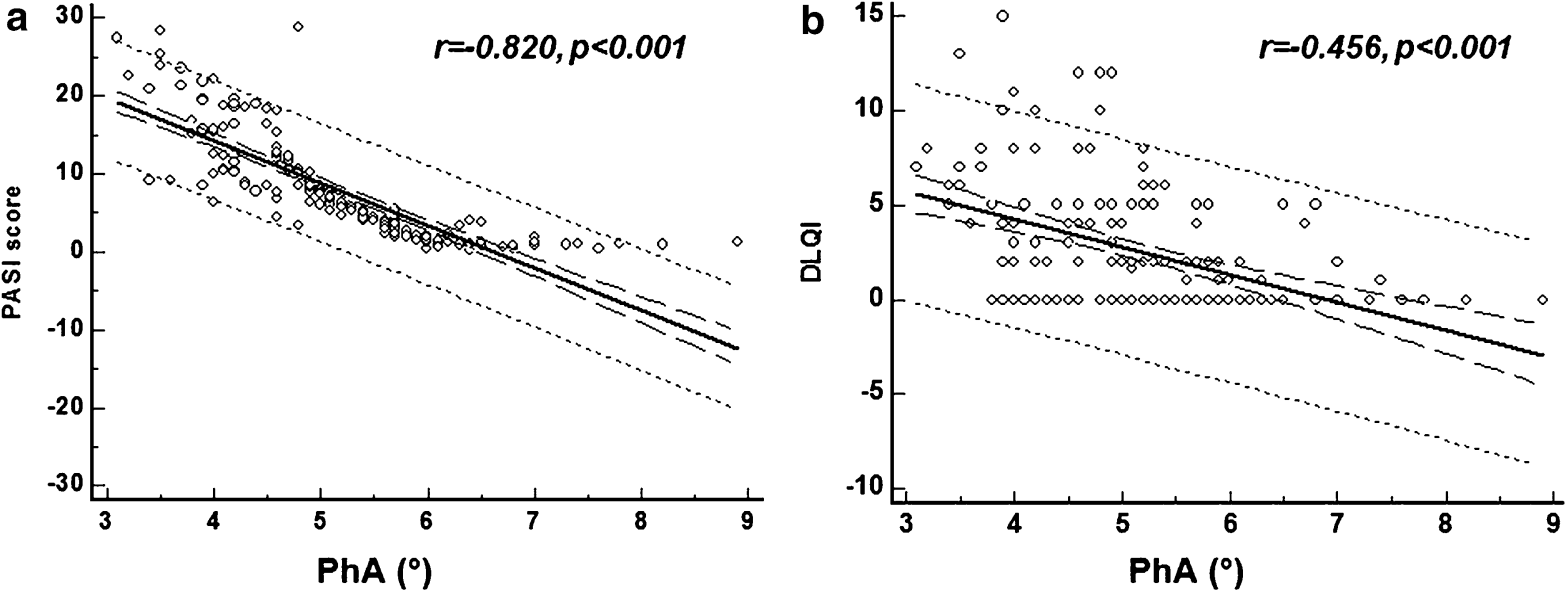
Correlation among PASI score and DLQI with the PhA. There was a significant negative association among PASI score (**a**) and DLQI (**b**) with the PhA. Correlations among variables were analyzed using Pearson *r* correlation coefficient. *PASI* psoriasis area and severity index, *DLQI* dermatology life quality index, *PhA* phase angle

Among psoriatic patients, PASI score and DLQI evidenced also significant positive correlations with anthropometric measures and metabolic profile, while between bioelectrical variables there were positive correlations with R and negative with Xc (Tables 3, 4). In addition, we found that PhA was significantly and negatively associated with either PASI score or DLQI (r = −0.810, p < 0.001 and r = −0.424, p < 0.001, respectively). The sample size used gives high power to test *r* (at p = 0.05 and r = −0.827; power 99 %). These negative associations remained significant after correction for BMI (Fig. 4a) and DLQI (Fig. 4b).

**Table 3:** Correlations among PASI score and study variable in psoriatic patients

Parameters	Simple correlation		Adjusted for BMI	
	PASI score		PASI score	
	r	*p* value	r	*p* value
Age (years)	0.129	0.083	0.147	0.057
DLQI	0.632	*<0.001*	0.612	*<0.001*
Anthropometric variables				
BMI (kg/m^2^)	0.306	*<0.001*	–	–
WC (cm)	0.405	*<0.001*	0.272	*<0.001*
Metabolic profile				
SBP (mmHg)	0.219	*0.003*	0.162	*0.036*
DBP (mmHg)	0.177	*0.017*	0.110	0.153
Glucose (mg/dl)	0.181	*0.015*	0.109	0.158
Total cholesterol (mg/dl)	0.398	*<0.001*	0.499	*<0.001*
HDL cholesterol (mg/dl)	−0.368	*<0.001*	−0.339	*<0.001*
LDL cholesterol (mg/dl)	0.489	*<0.001*	0.462	*<0.001*
Triglycerides (mg/ml)	0.503	*<0.001*	0.474	*<0.001*
AST (U/l)	0.203	*0.006*	0.131	0.089
ALT (U/l)	0.184	*0.014*	0.096	0.215
MetS (n. parameters)	0.450	*<0.001*	0.362	*<0.001*
CRP levels (ng/ml)	0.430	*<0.001*	0.383	*<0.001*
Bioelectrical variables				
R (Ohm/m)	0.312	*<0.001*	0.414	*<0.001*
Xc (Ohm/m)	−0.587	*<0.001*	−0.529	*<0.001*

Simple and partial correlation among PASI score, age, DLQI, anthropometric variables, metabolic profile and bioelectrical variables. There was a significant positive correlation among PASI score and DLQI, BMI, WC, SBP, DBP, glucose, lipid and hepatic profile, MetS and R; moreover there was a significant negative association with HDL-cholesterol and Xc. After adjustment for BMI, the association among PASI score and DBP, glucose, AST and ALT, were lost

Correlations among variables were analyzed using Pearson *r* or Spearman’s *rho* correlation coefficients. A *p* value in italic denotes a significant difference (p < 0.05)

*PASI* psoriasis area and severity index, *DLQI* dermatology life quality index, *BMI* body mass index, *WC* waist circumference, *SBP* systolic blood pressure, *DBP* diastolic blood pressure, *HDL* high-density lipoprotein, *LDL* low-density lipoprotein, *AST* aspartate aminotransferase, *ALT* alanine aminotransferase, *MetS* metabolic syndrome, *CRP* c-reactive protein, *R* resistance, *Xc* reactance

**Table 4:** Correlations among DLQI and study variable in psoriatic patients

Parameters	Simple correlation		Adjusted for BMI	
	DLQI		DLQI	
	r	*p* value	r	*p* value
Age (years)	0.128	0.086	0.117	0.131
Anthropometric variables				
BMI (kg/m^2^)	0.254	*0.001*	–	–
WC (cm)	0.264	*<0.001*	0.087	0.258
Metabolic profile				
SBP (mmHg)	0.247	*0.001*	0.202	*0.009*
DBP (mmHg)	0.136	0.069	0.094	0.224
Glucose (mg/dl)	0.154	*0.039*	0.105	0.174
Total cholesterol (mg/dl)	0.221	*0.003*	0.285	*<0.001*
HDL cholesterol (mg/dl)	−0.200	*0.007*	−0.155	*0.044*
LDL cholesterol (mg/dl)	0.282	*<0.001*	0.245	*0.001*
Triglycerides (mg/ml)	0.387	*<0.001*	0.301	*<0.001*
AST (U/l)	0.106	0.156	−0.007	0.930
ALT (U/l)	0.117	0.117	0.029	0.706
MetS (n. parameters)	0.280	*<0.001*	0.186	*0.016*
CRP levels (ng/ml)	0.357	*<0.001*	0.312	*<0.001*
Bioelectrical variables				
R (Ohm)	0.082	0.273	0.150	*0.045*
Xc (Ohm)	−0.404	*<0.001*	−0.336	*<0.001*

Simple and partial correlation among DLQI and age, anthropometric variables, metabolic profile and bioelectrical variables. There were significant positive correlations among BMI, WC, SBP, glucose, lipid profile and MetS; moreover there were significant negative associations with HDL-cholesterol and Xc. After adjustment for BMI, the association among DLQI and WC and glucose, were lost. Correlations among variables were analyzed using Pearson *r* or Spearman’s *rho* correlation coefficients. A *p* value in italic denotes a significant difference (p < 0.05)

*DLQI* dermatology life quality index, *BMI* body mass index, *WC* waist circumference, *SBP* systolic blood pressure, *DBP* diastolic blood pressure, *HDL* high-density lipoprotein, *LDL* low-density lipoprotein, *AST* aspartate aminotransferase, *ALT* alanine transaminase, *MetS* metabolic syndrome, *CRP* c-reactive protein, *R* resistance, *Xc* reactance

In addition, the PhA was significantly and negatively associated with number of parameters of MetS and CRP levels, independently of BMI levels (p < 0.001) in psoriatic patients only. BMI, PhA, PASI score and DLQI bivariate proportional odds ratio models for presence/absence of MetS were reported in Table 5. Among psoriatic patients, a small PhA was significantly associated with high odds of MetS presence (OR 5.87, 95 % CI 5.07–6.79), with a smaller PhA influencing the presence of MetS. At model fittings, the PhA was the major index value for diagnosis of MetS compared with BMI, and with the measures of clinical severity and quality of life, also after adjustment for BMI.

**Table 5:** Bivariate proportional odds ratio models performed to assess the association of MetS (Yes/No) and BMI, PhA, PASI score and DLQI in psoriatic patients

	Odds	*p* value	95 % CI	
*Model 1 BMI (kg/m* ^2^ *)*		*<0.001*		
MetS Yes	2.23		1.93–2.58	
MetS No	1.21		1.05–1.40	
*Adjusted for BMI*				
*Model 2 PhA (°)*		*<0.001*		
MetS Yes	5.87		5.07–6.79	
MetS No	0.46		0.39–0.53	
*Model 3 PASI score*		*<0.001*		
MetS Yes	2.20		1.90–2.55	
MetS No	1.23		1.06–1.42	
*Model 4 DLQI*		*<0.001*		
MetS Yes	2.23		2.03–2.73	
MetS No	1.15		0.99–1.33	

	Model 1 BMI (kg/m^2^)	Model 2 PhA (°)	Model 3 PASI score	Model 4 DLQI	
Model value fittings					
AIC	200.2	231.2	195.9	227.1	
R^2^ adj	0.06	0.01	0.17	0.04	

Bivariate proportional odds ratio models performed to assess the association of MetS (yes/no) and BMI (model 1), PhA (model 2), PASI score (model 3) and DLQI (model 4) in psoriatic patients. In model 2, the model fitting is better with presence of MetS than absence of MetS: small values of the PhA influence the presence of MetS among psoriatic patients. At model fittings, the PhA is a major index value for diagnosis of MetS. AIC value fitting and R^2^ are higher and lower, respectively, than the AIC value fitting and R^2^ of BMI, PASI score and DLQI. A *p* value in bold type denotes a significant difference (p < 0.05)

*MetS* metabolic syndrome, *BMI* body mass index, *PhA* phase angle, *PASI* psoriasis area and severity index, *DLQI* dermatology life quality index, *AIC* akaike information criterion, *CI* confidence interval

At multiple regression analysis, among PASI score, BMI, and number of parameters of MetS (model 1) or DQLI (model 2), the PhA well predicted the severity of psoriasis expressed by PASI score; among DLQI, BMI, number of parameters of MetS (model 1) or DQLI (model 2), the PhA and MetS well predicted the quality of life measured by DLQI (Table 6).

**Table 6:** Multiple Regression analysis models (stepwise method) with the PhA as dependent variables to estimate the predictive value of: (i) BMI, MetS parameters, and PASI score; (ii) BMI, MetS parameters, and DLQI

Multiple regression analysis				
Parameters	r^2^	*β*	t	*p* value
*Model 1*				
PASI score	0.670	−0.820	−19.10	*<0.001*
Variables excluded				
BMI, MetS parameters				
*Model 2*				
DLQI	0.204	−0.380	−5.70	*<0.001*
MetS parameters	0.269	−0.274	−4.11	*<0.001*
Variable excluded				
BMI				

Multiple linear regression analysis with the PhA as dependent variables were used to estimate the predictive value of PASI score BMI, MetS parameters and DLQI. In the model 1, the PhA was the major predictor of PASI score, while, in the model 2 DLQI and MetS parameters were the major predictor of the PhA. A *p* value in italic denotes a significant difference (p < 0.05)

*PASI* psoriasis area and severity index, *BMI* body mass index, *MetS* metabolic syndrome, *DLQI* dermatology life quality index, *PhA* phase angle

**Fig. 5:**
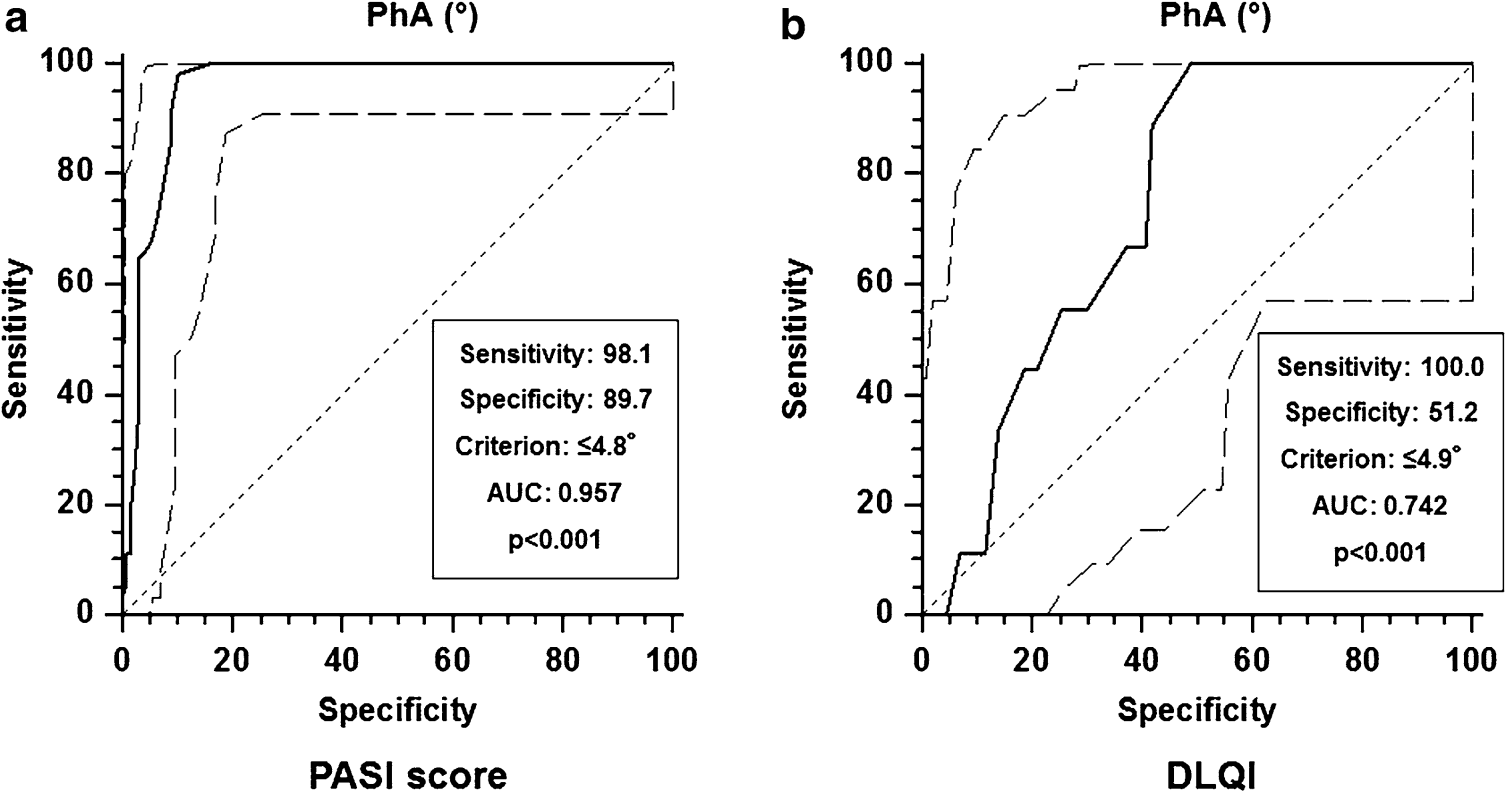
Receiver operating characteristic analysis (ROC) for predictive values of the PhA in detecting clinical severity and quality of life. Based on ROC curves, the most sensitive and specific cutoffs of the PhA to predict the highest PASI score (**a**) and the lowest DQLI (**b**) were ≤4.8° and ≤4.9°, respectively. *PhA* phase angle, *PASI* psoriasis area and severity index, *DQLI* dermatology life quality index, *ROC* receiver operating characteristic

ROC analysis for predictive values of the PhA in detecting clinical severity of psoriasis (PASI) and quality of life (DQLI) was reported in Fig. 5a, b, respectively. From the AUC the number of cases required for each group was set at 11 (Fig. 5b). Based on ROC curves, the most sensitive and specific cutoffs of the PhA to predict the highest PASI score and the lowest DQLI were ≤4.8° and ≤4.9°, respectively.

## Discussion

The results of this case–control, cross-sectional study report a novel association between PhA, a general indicator of cell membrane integrity, and psoriasis. In particular, our data well demonstrated that PhA was smaller in psoriatic patients than in healthy subjects, and that this difference is independently of gender. Moreover, the common association between PhA and age was not found in psoriatic patients. In addition, we found significant correlations between PhA with the clinical severity of psoriasis, expressed by PASI score and CRP levels, and the quality of life in these patients, independently of BMI. Based on ROC curve analysis, PhAs ≤ 4.8° and ≤4.9° identified psoriatic patients who have the highest clinical severity and the lowest quality of life, respectively.

Our data confirms the association between PhA and MetS in healthy subjects [30, 31], while we are not aware of any previous study examining this association in psoriatic patients. As expected, in both groups smaller PhAs were correlated with a higher degree of MetS. This association was independent of BMI in psoriatic patients only, suggesting the major role of the chronic systemic inflammation of psoriasis on PhA.

The PhA is a derived measure obtained from the relation between the BIA direct measures of R and Xc [14]. R is the opposition offered by the body to the electrical flow, and it is inversely related to the water and electrolyte content of tissues. Xc is the opposition to the electrical flow caused by the capacitance properties of the cell membrane, and variations can occur depending on its integrity, function, and composition [32]. The PhA is interpreted as an indicator of cellular health [18] and water distribution between the intra- and extracellular spaces [33]. Thus, the PhA is directly related to a low body cell mass [34] and to a high ECW/ICW ratio [35]. Different from the other parameters used to assess body composition measured by BIA, the PhA is considered to be valid also when the hydration status varies, as in obesity and chronic inflammation [19, 20, 36]. Small PhA was reported in patients with cancer [20], diabetes [21], and obesity [22], possibly because of a reduction in the mass of metabolically active cells [37]. In addition, the adjunctive effect on cell damage of the inflammatory status, commonly accompanying these conditions, should be considered [38].

Accordingly, the PhA has been proposed as a prognostic indicator in cancer [20] and as index for assessing catabolic state in diabetic patients [21]. Buscemi et al. [39] previously reported that a moderate osmotic effect of hyperglycaemia may induce a shift in ICW to ECW in diabetic patients, resulting in smaller PhA. This association has been recently confirmed by Dittmar et al. [21] who suggested the measurement of the PhA as independent prognostic markers of catabolic state and poor control in diabetic patients.

In our study, as expected, psoriatic patients had a worse metabolic profile as compared with their matched counterparts, with a positive correlation between MetS parameters and the severity of psoriasis [40]. In addition, we found also a negative correlation between MetS and the quality of life in these patients. Of interest, PhA, a non-invasive and bloodless measure, was a major index value for the diagnosis of MetS in our group of psoriatic patients. The identification of prognostic factors for psoriatic patients is important for the clinical management of the disease, for facilitates the assessment of the severity and prognosis of the disease, and the adequate monitoring of its clinical progress. To the best of our knowledge, there are no previous studies evidencing the association of the PhA with the MetS, the clinical severity and the quality of life in adult obese patients with psoriasis.

Our findings indicated that, unlike the healthy subjects [14, 18], in our group of psoriatic patients there was no evidence of sex-related differences in the PhA, nor PhA was associated with age. In line with the phase angle reference values stratified by age, sex, and BMI [15], higher values of PhA are expected among men because the PhA increases together with the muscle mass and the body cell mass [13, 16, 32, 41]. Furthermore, the PhA tends to decrease with age, as a function of the reduction of muscle mass and the influence of the alterations in the ICW/ECW ratio associated with aging [14, 42]. In that, our data are not in line with the study of Stobäus et al. [19] showing a significant influence of age and sex on the PhA in a large series of hospitalized patients in a retrospective analysis. This apparent contrast let us to speculate that the generalized inflammatory milieu characterizing psoriasis could likely hamper the negative effects of aging on PhA in psoriatic patients.

The inflammatory milieu could also account for the association between PhA and quality of life in our group of psoriatic patients. Sarcopenia, an aging-induced generalized decrease in muscle mass, strength, and function [43], is related to poor quality of life [44, 45]. In general, studies on body composition in inflammatory disease have shown a premature and ‘accelerated’ development of sarcopenia, especially in those with higher activity of disease and disability [46]. Muscle loss, linked to the increased expression of tumor necrosis factor (TNF)-α, is commonly reported in psoriasis [12, 47]. Sarcopenia as well as obesity sarcopenia are associated with a small PhA [16, 48]. A small PhA was used as a measure of low lean body mass in children with bowel inflammatory diseases [49]. Consequently, it is tempting to hypothesize that PhA could be determined by the combined effects of either the low grade inflammation associated with MetS and the systemic inflammatory status associated with psoriasis on the muscle mass, which may go unrecognized using BMI alone.

In summary, the major findings of this study were (i) psoriatic patients of both gender have smaller PhAs compared with healthy subjects, independently of age; (ii) PhA is independently and negatively associated with the clinical severity and the quality of life in psoriatic patients; (iii) PhA is associated with MetS independently of BMI, and represents a major index value for the diagnosis of MetS in psoriatic patients. Based on these findings, we hypothesize that the inflammatory milieu characterizing psoriasis could, on the one hand, account for the association of the PhA with MetS, the clinical severity, and the quality of life, independently of BMI and, on the other hamper the effect of age on the PhA.

Limitations of this study warrant some considerations. Firstly, the cross-sectional nature of this study did not allow to identify any causal association between psoriasis and PhA and to determine the prognostic value of PhA for predicting the MetS. Moreover, the suggested cut-off values of the PhA for diagnosing the clinical severity and the quality of life in our present study should be viewed with caution until results of studies in larger patient populations have become available to perform an appropriate cross-validation. Second, the association between psoriasis and PhA should be verified considering also the disease duration, and the detection of circulating markers of inflammation different from PASI and CRP levels, such as pro-inflammatory cytokines. Finally, expert Nutritionists are required for the assessment, execution and especially for interpretation of BIA measurements. However, this study has adequate statistical power and there were statistically significant differences in PhAs between psoriatic patients and healthy. In addition, in order to improve the power of the study, we applied very stringent inclusion criteria, such as a diagnosis of psoriasis lasting <6 months or the absence of any treatment for psoriasis.

## Conclusions

Overall, the findings of this study suggest that the PhA, a simple biophysical parameter, could be incorporated into routine clinical practice, as index of clinical severity, quality of life the and MetS in psoriatic patients, and marker of lean mass deficits, which may go unrecognized using BMI alone. Further studies are required, with larger and more diversified samples with respect to the severity and the duration of disease, to enable a more accurate assessment of PhA’s prognostic ability in patients with psoriasis and to understand the potential mechanisms by which chronic inflammation affects both PhA and MetS.

## Abbreviations

BMI: body mass index
BIA: bioelectrical impedance analysis
PhA: phase angle
R: resistance
Xc: reactance
PASI: psoriasis area and severity index
DLQI: dermatology life quality index
MetS: metabolic syndrome
WC: waist circumference
SBP: systolic blood pressure
DBP: diastolic blood pressure
HDL: high-density lipoprotein
LDL: low-density lipoprotein
AST: aspartate aminotransferase
ALT: alanine aminotransferase
CRP: c-reactive protein
